# RpkA, a Highly Conserved GPCR with a Lipid Kinase Domain, Has a Role in Phagocytosis and Anti-Bacterial Defense

**DOI:** 10.1371/journal.pone.0027311

**Published:** 2011-11-02

**Authors:** Tanja Y. Riyahi, Frederike Frese, Michael Steinert, Napoleon N. Omosigho, Gernot Glöckner, Ludwig Eichinger, Benoit Orabi, Robin S. B. Williams, Angelika A. Noegel

**Affiliations:** 1 Institute of Biochemistry I, Medical Faculty, Center for Molecular Medicine Cologne (CMMC), Cologne Excellence Cluster on Cellular Stress Responses in Aging-Associated Diseases (CECAD), University of Cologne, Köln, Germany; 2 Institute for Microbiology, Technical University Braunschweig, Braunschweig, Germany; 3 Leibniz-Institute of Freshwater Ecology and Inland Fisheries, Berlin, Germany; 4 School of Biological Sciences, Centre for Biomedical Sciences, Royal Holloway University of London, Egham, United Kingdom; French National Centre for Scientific Research - Université Aix-Marseille, France

## Abstract

RpkA (Receptor phosphatidylinositol kinase A) is an unusual seven-helix transmembrane protein of *Dictyostelium discoideum* with a G protein coupled receptor (GPCR) signature and a C-terminal lipid kinase domain (GPCR-PIPK) predicted as a phosphatidylinositol-4-phosphate 5-kinase. RpkA-homologs are present in all so far sequenced *Dictyostelidae* as well as in several other lower eukaryotes like the oomycete *Phytophthora*, and in the *Legionell*a host *Acanthamoeba castellani*. Here we show by immunofluorescence that RpkA localizes to endosomal membranes and is specifically recruited to phagosomes. RpkA interacts with the phagosomal protein complex V-ATPase as proteins of this complex co-precipitate with RpkA-GFP as well as with the GST-tagged PIPK domain of RpkA. Loss of RpkA leads to a defect in phagocytosis as measured by yeast particle uptake. The uptake of the pathogenic bacterium *Legionella pneumophila* was however unaltered whereas its intra-cellular replication was significantly enhanced in *rpkA^-^*. The difference between wild type and *rpkA^-^* was even more prominent when *L. hackeliae* was used. When we investigated the reason for the enhanced susceptibility for *L. pneumophila* of *rpkA^-^* we could not detect a difference in endosomal pH but *rpkA^-^* showed depletion of phosphoinositides (PIP and PIP_2_) when we compared metabolically labeled phosphoinositides from wild type and *rpkA^-^*. Furthermore *rpkA^-^* exhibited reduced nitrogen starvation tolerance, an indicator for a reduced autophagy rate. Our results indicate that RpkA is a component of the defense system of *D. discoideum* as well as other lower eukaryotes.

## Introduction

Receptors are known to play important roles in phagocytosis and immunity in mammals. For example, Fc receptors, mannose receptors and scavenger receptors all reside at the cell surface of professional phagocytes and trigger phagocytosis upon binding their specific ligand [1]. Other receptors play a major role in immunity processes, e.g. Toll-like receptors, which serve as recognition receptors of pathogen-associated molecular patterns (PAMPs). These receptors are also present on maturing phagosomes. They participate in analyzing the content of the phagosome , trigger immune reactions upon stimulation and may influence the association of phagosome-binding proteins, as well as the maturation state of the phagosome, although this is still under debate [2], [3]. In the professional phagocyte *D. discoideum* specific receptors for phagocytosis still remain unknown if they exist at all [4]. The same holds true for receptors which are involved in analyzing the phagosomal content. Until now only a few proteins have been identified in *D. discoideum* which are involved in phagocytosis and bacterial defense like the nine-transmembrane protein Phg1p or TirA a protein containing a Toll-Interleukin receptor domain [5], [6].

Macrophages as well as *Dictyostelium* phagocytose a number of bacteria, but not all of them are effectively destroyed. Pathogenic bacteria like *Listeria monocytogenes* and *Shigella flexneri* are specialized to escape the phagosome [7], [8], whereas others like *Mycobacterium tuberculosis* and *Legionella pneumonia*, staying inside the phagosome evade degradation by subverting maturation of the phagosome. One way to inhibit the phagosome maturation is to conceal its identity, which at least partially depends upon phosphoinositide composition of the membrane [9], [10], [11].

In recent years the social amoeba *D. discoideum* emerged as a suitable host to study infections with *L. pneumophila* [12], [13]. As a professional phagocyte feeding on a variety of bacteria, *D. discoideum* is an ideal macrophage model. The gram-negative bacterium *L. pneumophila* is the pathogenic agent of Legionnaire's disease. Upon inhalation of contaminated aerosols it hijacks pulmonary macrophages in human hosts by reprogramming their phagosomes to become *Legionella*-containing vacuoles (LCVs). In this niche the bacteria undergo replication which ultimately leads to destruction of the macrophages and eventually to the clinical picture of pneumonia. In the US ∼8,000-18,000 projected cases of hospitalized Legionnaires' disease occur per year [14]. Still, humans are only one possible host and a “dead end street” for the bacteria because transmission from human to human does not occur [15]. In fact, the primary targets of *L. pneumophila* are free living amoebae (FLA) in which the bacterium lives, divides and foremost is able to switch to a highly infectious mature intracellular form (MIF) which only occurs when grown in amoeba [16]. Inside its natural host, *L. pneumophila* is shielded from the surroundings and can survive even in environments usually hostile to bacteria, like artificial water supply systems [17], [18]. FLA colonize water systems where they pose a threat to human health by hosting *Legionella*. Thus, understanding the molecular aspects of *Legionella* infection in amoebae as well as amoebal defense mechanisms provides a clear and present research goal [18], [19], [20].

We report on RpkA, a seven-helix transmembrane protein with a GPCR signature and a C-terminal lipid kinase domain predicted as a phosphatidylinositol-4-phosphate 5-kinase (GPCR–PIPK) localized in internal membranes. The RpkA gene is expressed throughout development and its loss is associated with a developmental defect [21]. The results presented here show that RpkA is specifically transported to maturing phagosomes with similar kinetics as V-ATPase and interacts with this protein complex. The overall pH however remains unaffected in the *rpkA^-^*. Loss of RpkA leads to a phagocytosis defect and results in an enhanced survival of *L. pneumophila* which could originate from a reduced phosphoinositide turnover and/or a reduced autophagy rate, making RpkA a component of the defense system of *D. discoideum*.

## Results

### RpkA homologs are highly conserved in lower eukaryotes

In our initial studies we found RpkA-related proteins in the genomes of *Phytophthora sojae* and *P. ramorum* each encoding twelve RpkA homologs [22]. Recently additional genome data became available which allow a more detailed assessment of the occurrence of RpkA homologs during evolution. We have identified RpkA homologs in the closely related *D. purpureum* [23] as well as in *D. fasciculatum* and *Polysphondylium pallidum* which belong to different groups of the dictyostelids [24], [25]. *D. purpureum* and *D. fasciculatum* harbor one *rpkA* gene each like *D. discoideum*, *P. pallidum* has two copies. The RpkAs in dictyostelids are highly homologous and share between 44 and 58% amino-acid identity (Table 1).

**Table 1:** Orthologues of RpkA in lower eukaryotes.

Organism	Accession #	# of aa	% identity	p-value	comment
*D. purpureum*	EGC30811	817	58	2e-129	
*P. pallidum*	EFA86763	671	47	1e-113	Gene prediction only partially correct
*P. pallidum*	EFA77626	437	49	8e-92	Gene prediction likely incomplete
*D. fasciculatum*	DFA_05466|31431	544	44	1e-105	Gene prediction likely incomplete
*Capsaspora owczarzaki*	EFW45007	854	25	4e-56	
*Phytophthora infestans*	EEY57162	934	25	1e-54	*P. infestans* harbours 13 paralogues in total
*Albugo laibachii Nc14*	CCA24965	1016	27	1e-20	
*Acanthamoeba castellanii*	http://www.hgsc.bcm.tmc.edu/microbial-detail.xsp?project_id=163Contigs19320_4087_14119: bases 11,600 - 14,637	>486	33–63	3e-42	No gene prediction available
*Amphimedon queenslandica*	XP_003387269	824	25	5e-51	

Genbank accession numbers are provided except for *D. fasciculatum* where the DictyBase (http://dictybase.org/) and *A. castellanii* where the contig IDs of the genome sequencing website are given.

RpkA homologs are also present in other more distantly related species such as in the amoebozoa *Acanthamoeba castellani*, in *Capsaspora owczarzaki*, an amoeboid symbiont of a pulmonate snail [26], in *Albugo laibachi*, a blister rust parasitic to *Arabidopsis thaliana* and in the sponge *Amphimedon queenslandica* [27] (Table 1). *Phytophthora*, *Albugo*, *Capsaspora* and *Amphimedon* are opisthokonts whereas dictyostelids and *Acanthamoeba* are not. Thus, RpkA is a phylogenetically ancient protein, which is also present in ancient animals, the sponges, but seems to be absent in higher eukaryotes.

### RpkA-GFP is present on acidic endosomal vesicles, on phagosomes and co-localizes with V-ATPase

Previously we reported that carboxy-terminal GFP-tagged fusions of RpkA from *D. discoideum* (RpkA-GFP) localize to intracellular vesicles [21]. To exclude that the GFP-tag influenced the subcellular localization, we produced a variant harboring a C-terminal HA-tag (RpkA-HA). RpkA-HA was absent from the plasma membrane and present on intracellular vesicles of different size (Figure S1A) resembling the distribution previously reported for RpkA-GFP [21]. Furthermore, RpkA-HA completely rescued the developmental phenotype of the mutant (Figure S1B). We thus conclude that the C-terminal tag is unlikely to interfere with the function or distribution of RpkA.

**Figure 1:**
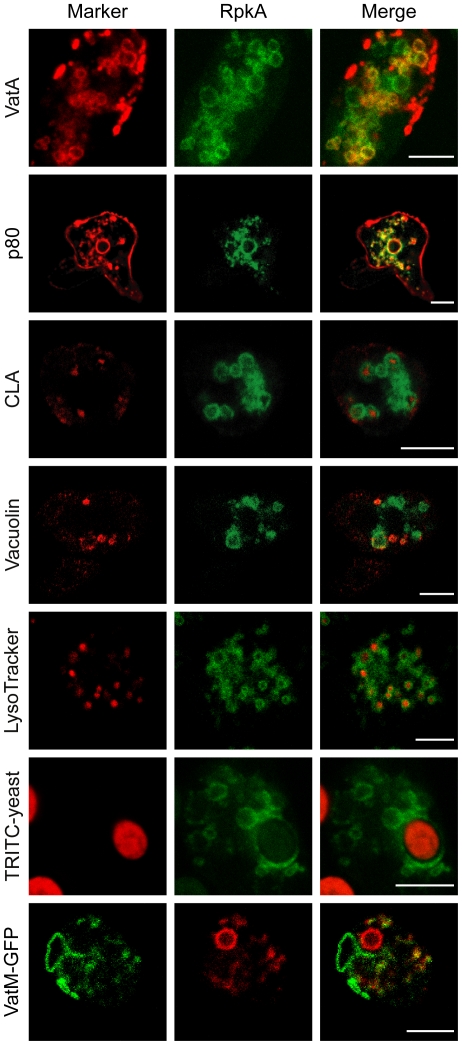
Localization of RpkA-GFP. RpkA-GFP expressing AX2 cells fixed with methanol were labeled with monoclonal mouse antibodies against subunit A of the vacuolar H^+^-ATPase, VatA (mAb 221-35-2), the putative copper transporter p80 (mAb H161), the common lysosomal antigen CLA (mAb 221-450-6) and the post-lysosomal marker vacuolin (mAb 263-79-2). As secondary antibody a goat-anti-mouse-IgG conjugated with Alexa 568 was used. Presence of RpkA-GFP in living AX2 cells on acidic compartments was identified by LysoTracker-Red. RpkA-GFP expressing cells were incubated for 15 min with TRITC-labeled *S. cerevisiae* and fixed with methanol. *rpkA^−^* cells expressing VatM-GFP and RpkA-RFP. Images were collected using the LSM confocal laser scanning microscope. Scale bar, 5 µm.

In *D. discoideum* the V-ATPase is part of the contractile vacuole, an organelle which is responsible for osmoregulation, as well as of acidic endosomes. Since we detected RpkA-GFP in the phagosomal membrane upon incubation of cells expressing RpkA-GFP with TRITC labeled yeast we evaluated the possible co-localization of RpkA-GFP with VatA (Figure 1, Figure S1A). Additionally, we co-expressed VatM-GFP, the membrane-spanning subunit of the V-ATPase complex, together with RpkA-RFP and observed areas of distinct co-localization in both cases (Figure 1). Consistent with these findings RpkA-GFP containing membranes enclose LysoTracker positive compartments which have an acidic pH, namely late endosomes, lysosomes and maturing phagosomes. Furthermore, the common lysosomal antigen CLA, a carbohydrate epitope detected by mAb 173–185-1[28], is present in several RpkA-GFP positive vesicles. Moreover, RpkA has recently been detected as part of the phagosome in a proteomic approach [29]. Little co-localization was observed between RpkA-GFP and vacuolin stained compartments, that represent post-lysosomal endosomes of neutral pH [30]. In contrast, there was a high degree of co-localization on internal membranes with p80, a putative copper transporter known to reside at the plasma membrane as well as throughout the whole endocytic transit (Figure 1) [31]. Thus, we conclude that RpkA is not a component of the plasma membrane but is rather present on phagosomes and a subpopulation of vesicles that are frequently acidic as well as positive for p80 and to some degree for V-ATPase.

### RpkA is recruited to phagosomes

**Figure 2:**
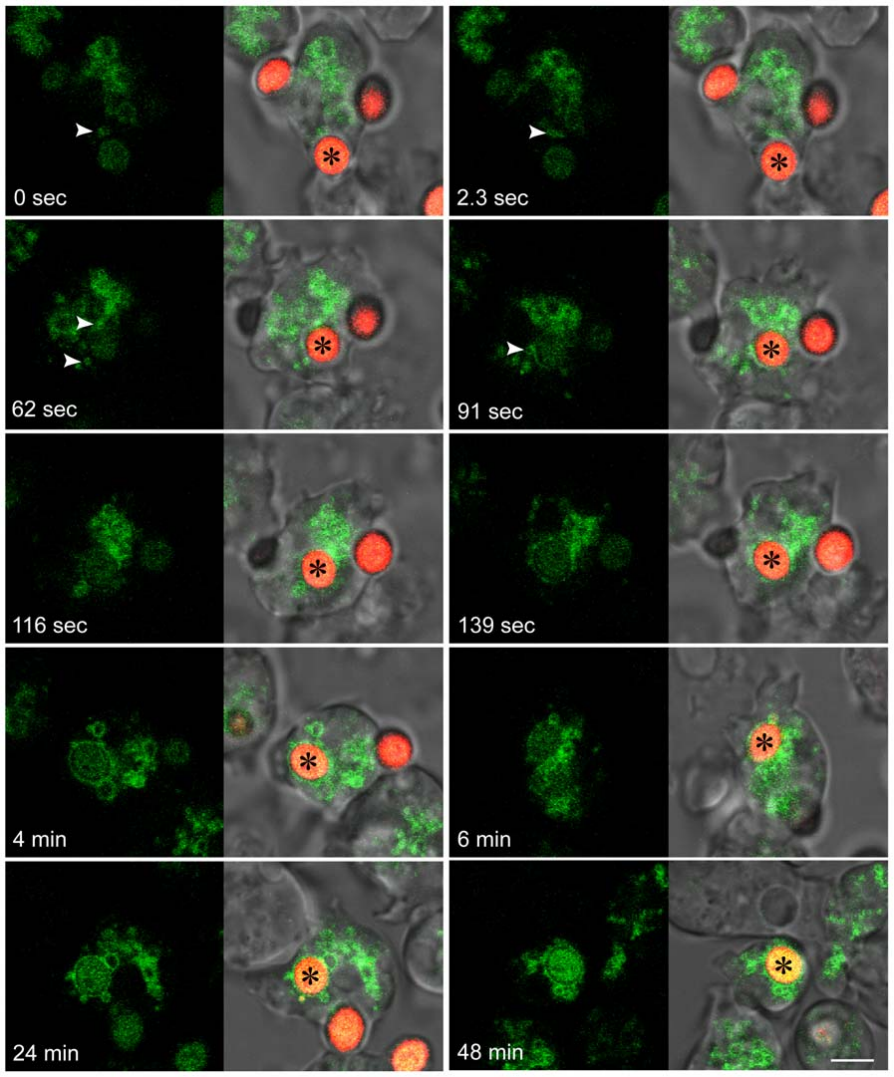
Recruitment of RpkA-GFP to the phagosomal membrane. Live cell analysis of TRITC-labeled *S. cerevisiae* uptake by AX2. Images were collected every 2.3 sec. Asterisks mark the yeast cell (red) which is taken up, arrowheads indicate RpkA-GFP containing vesicles (green) which are delivered to the maturing phagosome. Bar, 5 µm.

Since RpkA-GFP locates to phagosomes, we analyzed the timing of RpkA-GFP association with the phagosome during the uptake of yeast particles. Specifically, we wanted to determine whether the protein becomes part of the plasma membrane as a component of the phagocytic cup or if it is acquired later during maturation of the phagosome. AX2 (wild type) cells expressing RpkA-GFP were incubated with TRITC-labeled yeast and progress of phagocytosis was analyzed by confocal microscopy. We observed RpkA-GFP on vesicles of different diameters. These vesicles can be highly dynamic and approach the plasma membrane region forming the phagocytic cup, but they do not detectably fuse with the phagocytic cup (Figure 2, 0 and 2.3 sec, white arrow heads). From 91 sec onward RpkA-GFP is detectable in the phagosomal membrane. After approximately 60 seconds (Figure 2, 62 and 91 sec) RpkA-GFP containing vesicles (white arrow heads) start to fuse with the phagosome, thus suggesting a mechanism of directed RpkA-GFP delivery to the phagosomal membrane. A similar mode has been described for the V-ATPase [32]. At later time points RpkA-GFP staining is enhanced and the protein remains on the phagosome until the end of the image recordings (48 min). Thus, RpkA is specifically acquired by the phagosome during its maturation process.

### Loss of RpkA leads to a reduced phagocytosis rate

**Figure 3:**
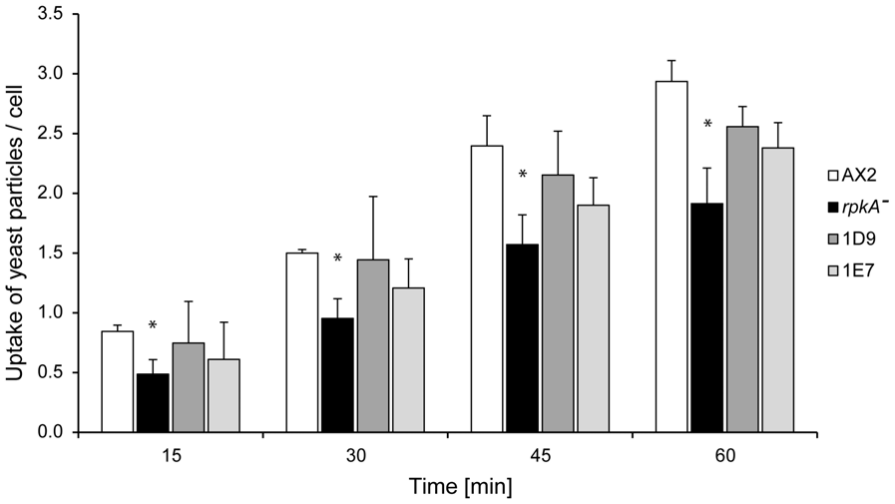
Phagocytosis of yeast cells. AX2 , *rpkA^−^* cells, and *rpkA^−^* rescue strains 1D9 and 1E7 expressing RpkA-HA were incubated with TRITC-labeled yeast cells and fixed at the indicated time points. The number of phagocytosed yeast particles per cell was quantified at the indicated time points. Results are provided for three independent experiments ± SE, *P<0.05.

To study the impact of RpkA on phagocytosis we quantified the uptake of yeast cells in AX2, *rpkA^−^*, and *rpkA^−^* expressing RpkA-HA (*D. discoideum rpkA^−^* rescue strains 1D9 and 1E7) over time using TRITC-labeled yeast. We found that the uptake of yeast cells in *rpkA^−^* cells was reduced at every time point when compared with wild type cells. On average *rpkA^-^* cells had taken up less than two yeast particles at 45 or 60 min (Figure 3). Also, after 45 min *rpkA^−^* cells did not take up any further yeast cells, whereas AX2 cells engulfed one more cell on average. The rescue strains show an improved phagocytosis compared to *rpkA^−^*, incorporating ∼2.3 yeast particles per cell compared to 1.5 for *rpkA^−^* at 60 min. However they reached only ∼74% of the wild type level which could be due to differing levels of RpkA-HA protein.

### Loss of RpkA affects survival of *Legionella*

Since RpkA is a component of phagosomal membranes which is acquired during the maturation process of the phagosome we wanted to know if RpkA has an impact on innate immunity related aspects like infection with *L. pneumophila* or autophagy.

**Figure 4:**
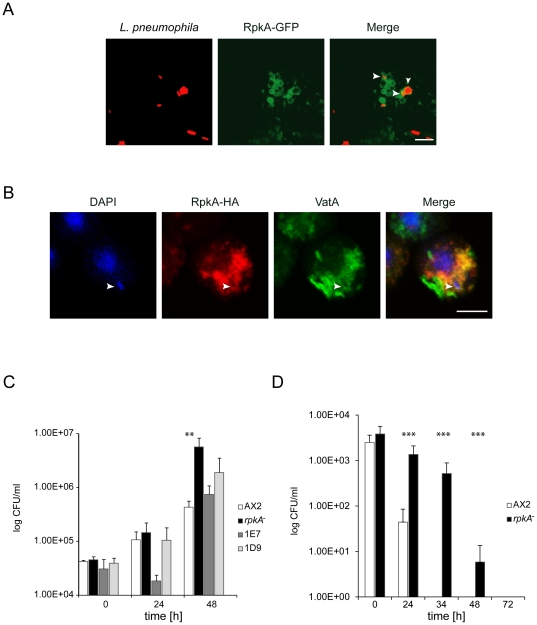
RpkA affects *L*. *pneumophila* infection. **(A)** **RpkA is present on vesicles containing heat-killed** ***L. pneumophila*** **.** AX2 cells expressing RpkA-GFP were incubated with heat killed rhodamine-labeled *L. pneumophila* for 10 min prior to fixation. The arrowhead points to a bacterium surrounded by RpkA labeled membrane. Bar, 5 µm. **(B) RpkA and VatA are present on vesicles containing living wild type** ***L. pneumophila*** **.** *rpkA^−^* cells expressing RpkA-HA (rescue strain 1E7) were incubated with living *L. pneumophila* and fixed after 1 h. Bacteria were visualized with DAPI, RpkA-HA with mAb 3F10 against the HA-tag and goat-anti-rat-IgG conjugated to Alexa 568 as secondary antibody. VatA was visualized with mAb 221-35-2 and goat-anti-mouse-IgG conjugated with Alexa 488 as secondary antibody. Arrowhead, V-ATPase and RpkA-HA positive vesicle containing *L. pneumophila*. Bar, 5 µm. **(C) Loss of RpkA leads to elevated titers of** ***L. pneumophila*** **.** AX2, *rpkA^-^* and the rescue strains 1D9 and 1E7 were infected with *L. pneumophila* for 3 h. Not ingested bacteria were removed and viable internal *L. pneumophila* were quantified (0 h). The quantification was done also at time points 24 and 48 h post infection. CFU, colony forming units. Results are provided for four experiments done in triplicates ± SD, **P<0.01. **(D) Infection with** ***L. hackeliae*** **leads to reduced clearing in** ***rpkA^−^*** **.** AX2 and *rpkA^−^* were infected with *L. hackeliae* for 3 h. Non ingested bacteria were removed and viable internal *L. hackeliae* were quantified (0 h). The quantification was done also at time points 24, 34, 48 and 72 h post infection. CFU, colony forming units. Results are provided for four experiments done in triplicates ± SD, ***P<0.001.

To get insight into a possible role of RpkA in *L. pneumophila* infection, we investigated whether dead TRITC-labeled *L. pneumophila* co-localize with RpkA positive phagosomes. Indeed, after incubation of AX2 cells expressing RpkA-GFP with rhodamine-labeled *L. pneumophila*, bacteria are found in phagosomes positive for RpkA-GFP (Figure 4A). Next we tested if live *L. pneumophila* are taken up into RpkA-positive vesicles. Therefore we incubated rescue strain 1E7 expressing RpkA-HA with unlabeled wild type *L. pneumophila*, fixed and stained for the HA-tag and VatA. We observed that whenever a *L. pneumophila* containing phagosomes was positive for RpkA-HA it was also positive for VatA and vice versa (Figure 4B). Next we wanted to know if the loss of RpkA influences the uptake and/or replication of *L. pneumophila* by carrying out infection studies with live *L. pneumophila*. Wild type, *rpkA^−^* and rescue strains 1D9 and 1E7 were infected with *L. pneumophila*. After removal of extracellular bacteria, internalized *Legionella* were quantified. The quantification was done at 0h, 24h and 48h post infection. No initial difference was seen for uptake of bacteria between strains (Figure 4C, 0h). After 48h the *L. pneumophila* content in *rpkA^−^* was 13 times higher than in AX2 (Figure 4C, 48 h). The rescue strains again showed an intermediate behavior. Thus the loss of RpkA does not influence the uptake of *L. pneumophila*, but the engulfed bacteria reach significantly higher titers in the absence of RpkA.

This difference becomes even more prominent if *L. hackeliae* is employed which is less virulent compared with *L. pneumophila* [33]. In human macrophages *L. hackeliae* replicates and causes pneumonia, whereas in amoebae it does not replicate and is killed. In AX2 within 34 hours the killing of *L. hackeliae* is completed, whereas in *rpkA^−^* it is significantly delayed and bacteria are still alive after 48 hours (Figure 4D).

### RpkA-GFP interacts with the V-ATPase complex

**Figure 5:**
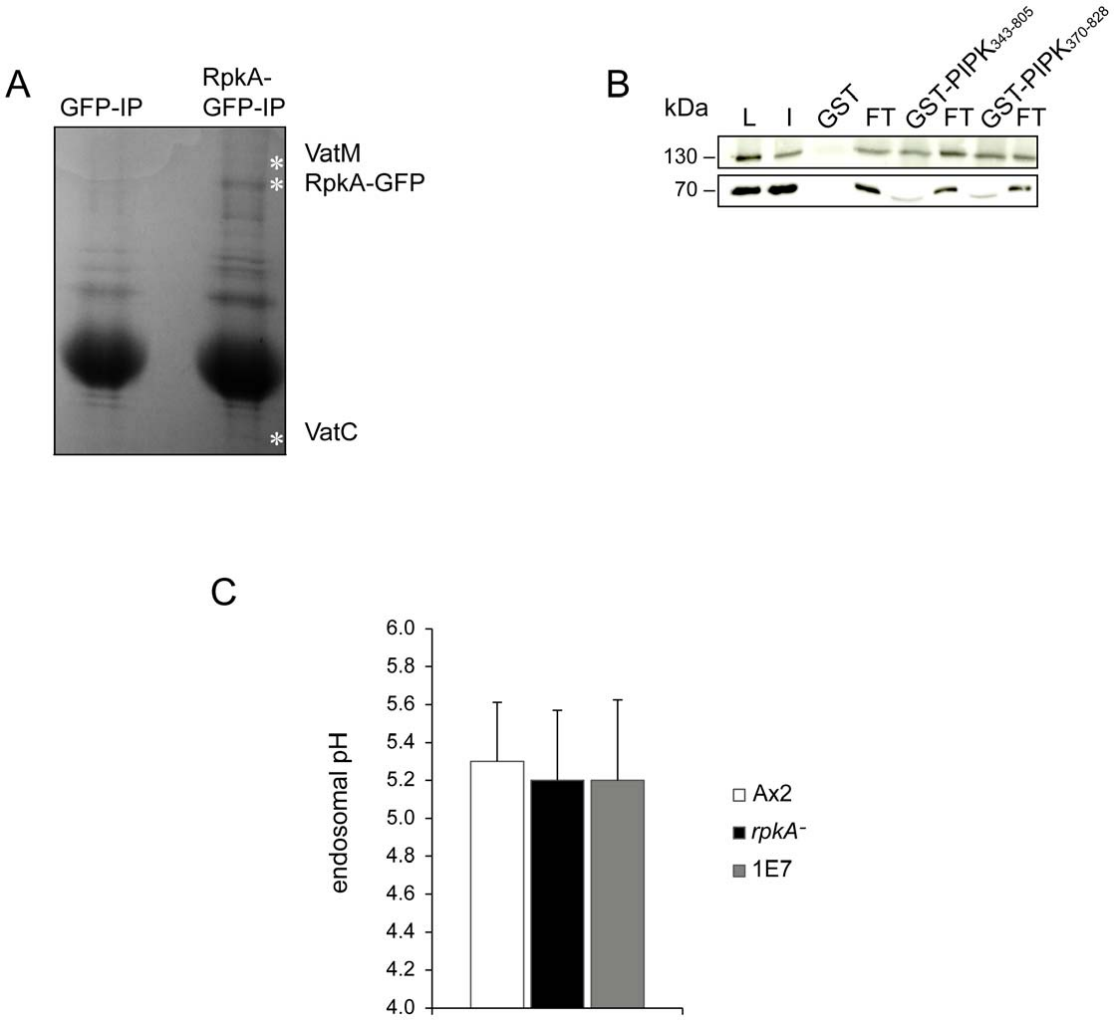
V-ATPase interacts with RpkA but average endosomal pH is uneffected in *rpkA^-^*. **(A) V-ATPase co-immunoprecipitates with RpkA-GFP.** Polyclonal GFP-antibodies were used for immunoprecipitation of either RpkA-GFP or GFP as a control. Proteins were separated by SDS-PAGE and stained with Coomassie Blue. VatM and VatC were detected as co-precipitates with RpkA-GFP by mass spectrometry. The positions of VatM, RpkA-GFP and VatC are indicated by white asterisks. **(B) VatA and VatM-GFP specifically co-precipitate with GST-PIPK**_**343 – 805**_ **and GST-PIPK**_**370 – 828**_. GST, GST-PIPK_343 – 805_ and GST-PIPK_370 – 828_ were incubated with a lysate of *rpkA^-^* cells expressing VatM-GFP. Western blots of the pull down were probed using GFP-specific mAb K3-184 to detect VatM-GFP at 130 kDa and VatA specific mAb 223-35-2 to detect VatA at 70 kDa. The western blot shows the lysate of 2×10^5^ cells (L), 10 µl of input (I), the pull downs (GST, GST-PIPK_343 – 805_ and GST-PIPK_370 – 828_) and respective flow through fractions (FT). In the lower panel a Coomassie-stained gel of the pull downs is shown. **(C) Endosomal pH is unaffected in** ***rpkA^−^*** **.** AX2, *rpkA^−^* and rescue strain 1E7 were incubated for 3 h with FITC-dextran as a pH probe. Then the excitation ratio at 495 nm/450 nm was measured and the endosomal pH was determined using a standard curve. Results are provided for quadruplet experiments ± SD.

Since RpkA is recruited to maturing phagosomes with the same kinetics as the V-ATPase complex we wanted to investigate if RpkA not only co-localizes with the V-ATPase but also interacts with this complex. Therefore, RpkA-GFP was immunoprecipitated from cell lysates, obtained proteins were separated by SDS-PAGE, and analyzed by mass spectrometry. We identified the subunits C and M of the V-ATPase in the immunoprecipitate (Figure 5A). The interaction with V-ATPase was further verified since we found VatA and VatM-GFP to co-precipitate with GST-PIPK_343 – 805_ (residues 343 – 805) and GST-PIPK_370 – 828_ (residues 370 – 828). Thus, we conclude that the PIPK domain of RpkA is responsible and sufficient for this interaction (Figure 5B).

### RpkA does not affect the overall endosomal pH

Since RpkA co-localizes and directly interacts with the V-ATPase, we investigated the influence of the loss of RpkA on the endosomal pH and incubated AX2, *rpkA^−^* and rescue strain 1E7 cells with FITC-Dextran and measured the endosomal pH. We observed an average pH of 5.3 for AX2 which is in agreement with published values [34]. The pH determined for *rpkA^−^* and 1E7 cells was similar (pH 5.2) which indicates that RpkA does not affect the overall endocytic pH (Figure 5C). Furthermore, *L. pneumophila* is known to inhibit the acquisition of V-ATPase which is responsible for establishing low pH values.

### RpkA affects the phosphoinositide metabolism of the cell

A characterization of the PIPK of RpkA as for any other PIPK of a GPCR-PIPK is lacking. Neither the substrates nor the products are known. The PI-kinase activity of RpkA might be one factor determining the resistance of *D. discoideum* towards *L. pneumophila* infection as phosphoinositides are also known to play a major role in *L. pneumophila* infection and previous work showed that the bacteria can subvert the host*'*s phosphoinositide turnover [35].

**Figure 6:**
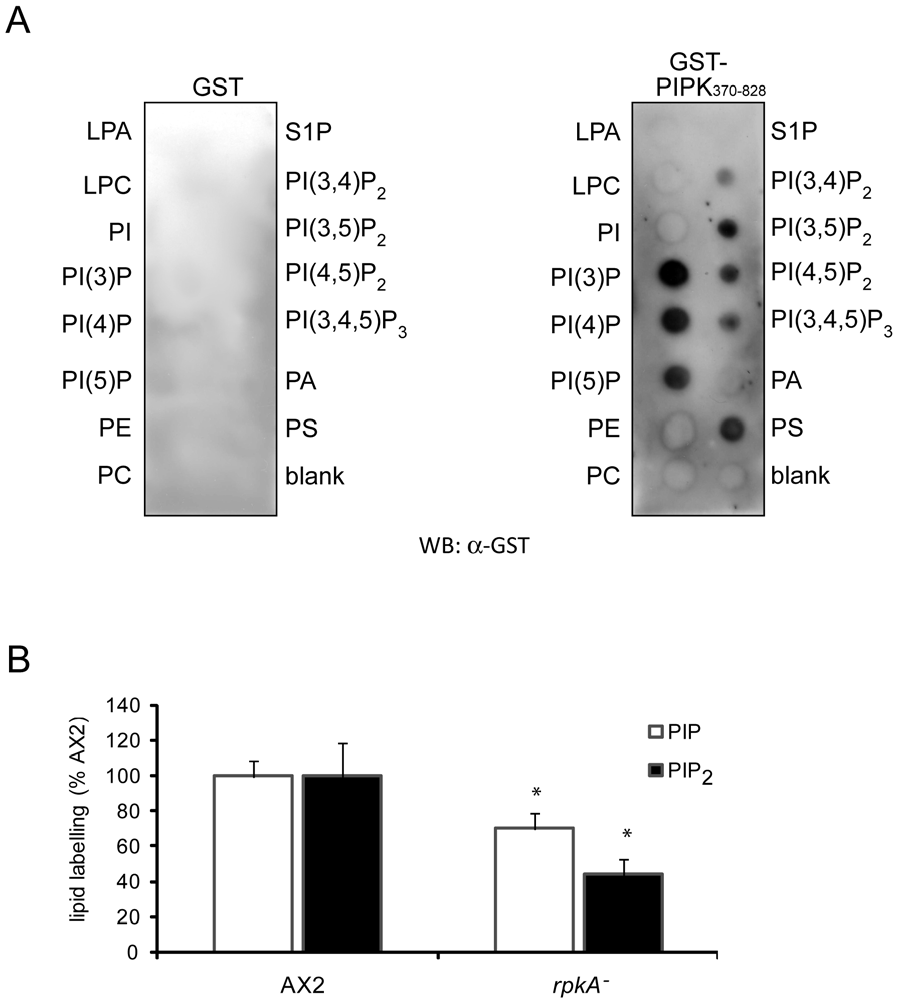
RpkA influences phosphoinositolphosphate turnover. (A) The PIPK_370 – 828_ domain binds to phosphoinositolphosphates. PIP-Strip-membranes were incubated over night with GST-PIPK_370 – 828_ (1 µg/ml) and with GST (1 µg/ml) for control. Binding was detected by incubation with polyclonal GST-antibodies. **(B) Loss of RpkA leads to reduced PIP and PIP_2_ levels.** Phosphoinositide turnover in AX2 and *rpkA^−^* cells was monitored by permeabilized cells, and labeling phospholipids with [γ-^32^P] ATP. Subsequently cells were lysed, phospholipids were extracted and separated by TLC, imaged using Typhoon phosphorimager, quantified with “ImageQuant” and normalized according to total lipids. Results are provided for triplicate experiments with duplicate samples ± SD, *P<0.05.

We wanted to approach the function of the PIPK of RpkA by in vitro and in vivo studies. First we tested the ability of the PIPK-domain of RpkA to bind to different phosphoinositides in vitro. In this study we expressed GST-PIPK_370–828_ in *E. coli* and performed a dot blot overlay assay to assess its binding ability to lipids (PIP-Strip), enabling the detection of the PI-kinase substrate [36]. GST-PIPK_370 –828_ bound preferentially to monophosphorylated PIs especially to PI3P and PI4P, consistent with a role in the generation of PIP_2_ from PI3P or PI4P (Figure 6A). GST-PIPK_370–828_ was also able to bind to phosphatidylserine. GST-PIPK_343–805_, on the contrary did not exhibit any lipid binding (data not shown), implicating that the last 23 residues are important for the lipid binding. With an isoelectric point of 4.0 these amino acids do not contribute to a general affinity to the negatively charged PIPs but they might stabilize the PIP binding domain of the PIPK. It is on the other hand as well conceivable but less probable that the additional 27 residues on the N-terminus of GST-PIPK_343 – 805_ inhibit the binding to the PIPs.

GST alone did not bind to any of the tested lipids indicating that the binding of the PIPK_370 – 828_ to the lipids is specific (Figure 6A).

### Loss of RpkA leads to reduced levels of phosphoinositides

To get an impression of the relevance of RpkA for the phosphoinositide metabolism of the cell we investigated the consequences of the loss of RpkA on phosphoinositide turnover by metabolic labeling of phospholipids using [γ-32P] ATP in vivo [37]. In *rpkA^−^* cells the turnover of monophosphorylated phosphoinositides (PIP) as well as bisphosphorylated phosphoinositides (PIP_2_) was reduced to 70% and 44% of the wild type (AX2) cells, respectively (Figure 6B). This was surprising because, assuming that RpkA is a PI4P5K, we expected that a loss of this enzyme would simply lead to an increase of the amount of PI4P (substrate) and a decrease of PI(4,5)P_2_ (product).

### Nitrogen starvation tolerance is reduced in *rpkA^−^* cells

**Figure 7:**
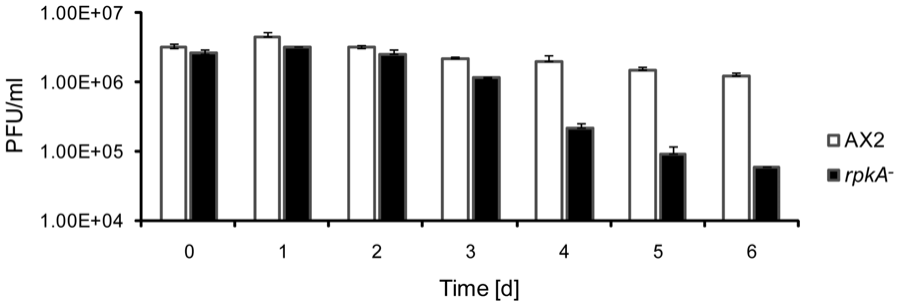
*rpkA^−^* cells show reduced tolerance against nitrogen starvation. AX2 and *rpkA^−^* cells were grown in FM-Medium for at least 5 generations, harvested during the exponential growth phase and resuspended at a density of ∼ 3×10^6^ cells/ml in FM medium lacking amino acids. At the indicated time points viability was determined by analyzing the ability to form colonies (PFU, plaque forming units) on bacterial lawns. Results are provided for duplicate experiments with duplicate samples ± SD.

Autophagy is a pathway which is involved in cell autonomous defense and helps to eliminate pathogenic bacteria that reside in the cytosol of the host cell through lysosomal degradation [38]. One of the earliest steps in autophagy is the activation of a specific class III phosphatidylinositol-3-OH kinase (PI3K) complex and the formation of phosphatidylinositol-3-phosphate (PI3P) in ER membranes which recruits proteins required for the formation of the autophagosome [39], [40]. Based on our findings that the PI metabolism is altered in the *rpkA^−^* strain and on the observation that mutants deficient for autophagy related genes show similar defects in development [41], [42], we assessed autophagy by testing the ability of the mutant to survive in the absence of an exogenous nitrogen source (nitrogen starvation assay) as autophagy is also strongly induced by nitrogen starvation [43], [44]. We found that under such conditions the cell numbers of AX2 stayed nearly constant over three to four days and then decreased slowly. In contrast, cell numbers of the *rpkA^−^* strain significantly decreased from day three onward. AX2 and *rpkA^−^* started with approximately the same cell density of ∼2.8×10^6^ cells/ml at day 0. After 6 days AX2 cultures had a density of 1.25×10^6^ cells/ml whereas *rpkA^−^* cultures had a 21-fold lower density (6×10^4^ cells/ml). Thus, the *rpkA^−^* mutant apparently cannot survive an extended period of nitrogen starvation (Figure 7).

## Discussion

GPCRs are generally known to be transported along the secretory pathway to the plasma membrane where they are active. Upon stimulation they can be internalized and can either cycle back to the plasma membrane or are sorted to late endosomes and can further be degraded within lysosomes.

We show that RpkA is delivered to phagosomes with similar kinetics as it has been published for the V-ATPase [32]. RpkA and the V-ATPase complex do not completely overlap in their localization, e.g. they do not co-localize in the contractile vacuole. In this compartment we only find the V-ATPase and RpkA is present on vesicles which are free of V-ATPase. However there are membranous compartments where both of them come together and can interact and one of these compartments is the maturing phagosome. To our knowledge RpkA is so far the first GPCR which is specifically associated with the maturing phagosome.

RpkA is also an interaction partner of the V-ATPase. The V-ATPase complex interacts with full length RpkA as well as with the PIPK _343 – 805_ and PIPK_370 – 828_ domain alone.

Loss of RpkA results in a reduced phagocytosis rate of yeast cells, whereas the uptake of *Legionella* does not differ in *rpkA^-^* and AX2 cells. This might be due to the difference in the uptake mechanism of yeast and *Legionella*. Both can be phagocytosed, however *Legionella* might be taken up primarily via macropinocytosis by *D. discoideum* [45]. Furthermore, the bacteria are taken up by coiling phagocytosis and foremost can induce their uptake since they can also infect HeLa cells and other nonprofessional phagocytes [46], [47], [48], [49].

Although the uptake of *L. pneumophila* is comparable in AX2 and *rpkA^−^* replication is significantly altered. *L. pneumophila* reaches 13 times higher numbers in *rpkA^−^* compared to AX2. The difference between wild type and mutant is even more intriguing regarding the less virulent *L. hackeliae* which survives far longer in *rpkA^−^*.

One reason for this effect may be that *L. pneumophila*, the most pathogenic *Legionella* strain, is able to manipulate even wild type *D. discoideum* so drastically that the difference between AX2 and a mutant is less obvious as it is in the case of a less harmful strain like *L. hackeliae*. Here the difference between AX2 and the mutant *rpkA^−^* becomes more prominent. AX2 is able to sustain the manipulation of *L. hackeliae*, whereas *rpkA^−^* has major problems to kill the bacteria. In macrophages which are more susceptible than *rpkA^−^ L. hackeliae* can even replicate [33]. The intermediate behavior of the rescue strains is most probably due to heterogeneous expression of RpkA-HA. Although 1E7 and 1D9 are of single clone origin the expression pattern varies within the cell population as observed by immunofluorescence.

Since RpkA interacts with the V-ATPase at the phagosome one of the reasons for the significantly higher *Legionella* titer in the *rpkA^−^* might be an elevated phagosomal or endosomal pH which we however did not detect. Although the overall endosomal pH is unaltered in *rpkA^−^* we cannot rule out that RpkA has an influence on early events of pH changes during phagocytosis like a slight retardation of the pH drop.

*L. pneumophila* inhibits normal phagosomal maturation by translocating effector molecules through the Dot/Icm type IV secretion system from the LCV into the host cell's cytosol [50]. However, the effector molecules responsible for the arrest of phagosomal maturation are still poorly understood. Recently, an effector protein has been discovered that inhibits the V-ATPase. If lysosomal proteins like the V-ATPase are inhibited solely by effector molecules secreted by *L. pneumophila* or if they are excluded from the LCV is still under debate [51], [52], [53], [54]. Phosphoinositides are instrumental in the deployment of the phagosomal antimicrobial defense [55]. They are involved in determining the identity of membranous compartments. The plasma membrane predominantly contains PI(4,5)P_2_, whilst the Golgi apparatus holds PI4P, late endosomes and lysosomes harbor PI(3,5)P_2,_ whereas early endosomes and phagosomes contain PI3P [56]. These phosphoinositides serve as anchors for specific proteins [57]. The phosphoinositide-bound proteins can enable the recruitment of further proteins or catalyze enzymatic reactions to establish a compartment-specific environment. Thus, the true nature of a phagosome can be concealed by a pathogen through interfering with the phosphoinositide composition by either prohibiting the acquisition or synthesis of PI3P, by promoting the degradation of PI3P or by binding PI3P with other proteins. This process of organelle disguise, by pathogens like *L. pneumophila,* has been designated ”identity theft” [10].

One example for such a phosphoinositide is PI3P, a marker of early phagosomes. *Legionella* can replicate better in *D. discoideum* cells upon inhibition of PI3Ks or when PI3K genes are disrupted or PI3Ks inactivated [45], [58] indicating *Legionella* can more easily camouflage the identity of the phagosome if less PI3P is present on the phagosome. *L. pneumophila* secretes several proteins that bind PI3P and PI4P. LpnE for instance, is a secreted protein that binds to PI3P, and is involved in inhibiting the acquisition of lysosomal markers and phagosomal maturation, while SidC binds to PI(4)P on the LCV membrane [35], [59].

Based on our findings that loss of RpkA significantly lowers the PIP and PIP_2_ levels in the mutant, phosphoinositide composition of the phagosome as well as vesicle trafficking towards the phagosome may be crucially altered in the mutant allowing facilitated establishment of a replicative vacuole. GST-PIPK_370 – 828_ is able to bind to phosphoinositides and phosphatidylserine as has been shown for other PIPKs [36], [60].

However, which phosphoinositide species are generated by GPCR-PIPKs has not been shown to date. It has been shown that the PIPKs of all the GPCR-PIPKs do not cluster with the known PIPKs ( Type I/II or III) but instead cluster in a new group (Type IV) [22]. It might well be that RpkA is directly involved in the generation of PIP and PIP2 as both species are diminished in *rpkA^−^*.

Autophagy is well known to play a role in innate immunity against cytoplasmic pathogens which are internalized into autophagosomes similar to mitophagy. The role of autophagy in infection with *Legionella* has been discussed controversially. Autophagy was initially assumed to be a favorable pathway exploited by *L. pneumophila* [61]. Later it was thought to play no role at all since in *D. discoideum* loss of ATG7 and ATG8 had no consequences for the replication of *L. pneumophila* [62]. Recently it has been reported that ATG7 and ATG8 localize to the LCV. The acquisition of these autophagy proteins to the LCV is elevated in mouse macrophages that are restrictive to *L. pneumophila* infection, in contrast to their acquisition in permissive mouse macrophages [63]. This suggests that autophagy is a mechanism of defense against the establishment of LCVs which is also supported by recent results in *D. discoideum* showing that loss of ATG9 leads to a lower clearance and higher replication rates of *L. pneumophila* [64].

Based on our findings RpkA is an endosomal GPCR-PIPK which is recruited to phagosomes during their maturation. It interacts with the V-ATPase and is involved in phagocytic processes. Its loss leads to a reduced resistance against the pathogen *L. pneumophila*. The protein seems to be ancient since it is conserved in several phylogenetically distant species. Till now RpkA homologs seem to be restricted to lower eukaryotes. The presence of an RpkA homolog in *A. castellani* is intriguing as this organism is an established primary host of *L. pneumophila*. Further analysis of the role of RpkA in *A. castellani* may provide us with tools for interfering with the environmental reservoir of *L. pneumophila*.

## Materials and Methods

### Growth, Transformation and Development

Cells were either grown on a lawn of *K. aerogenes* on SM agar plates or cultivated in shaking suspension (160 rpm) or in a submerged culture at 21–23°C in axenic medium [65]. Development was initiated by plating 5×10^7^ cells which were washed twice with Soerensen phosphate buffer (17 mM Na^+^/K^+^ phosphate, pH 6.0) on phosphate agar plates and monitored. Mutants were maintained in the presence of appropriate antibiotics (2–4 µg/ml G418 (Roche Applied Science) or 3–5 µg/ml blasticidin (MP Biomedicals Inc., Eschwege, Germany)). The following strains have been used; AX2-214 (wild type) [66], AX2 expressing GFP-tagged RpkA (RpkA-GFP, GFP fused to the C-terminus of RpkA) or HA-tagged RpkA (RpkA-HA, HA-tag fused to the C-terminus of RpkA), *rpkA^-^* [21] and *rpkA^-^* rescue strains 1E7 and 1D9 expressing RpkA-HA, AX2 expressing RFP-tagged RpkA (RpkA-RFP, mRFPmars [67] fused to the C-terminus of RpkA).

### Phagocytosis assays and *Legionella* infection

Phagocytosis was assayed on a substratum where the cells were allowed to settle on coverslips and yeast cells (∼20 yeast cells/*Dictyostelium* cell) were added. After the indicated times the cells were fixed in methanol, and embedded. Approximately 150 cells per strain and time point were analyzed for uptake of yeast particles in two independent experiments [68]. Infection with *L. pneumophila* was done as described with the exception that *L. pneumophila JR32* Phil was used for the assays [69]. *L. pneumophila JR32* Phil was cultured on BCYE plates (buffered charcoal yeast extract agar for 3 days at 37°C and a CO_2_ concentration of 5%. The bacteria were harvested in 1 ml of Soerensen buffer and adjusted to a density of 5×10^6^ colony forming units/ml.

*D. discoideum* cells of a 3 day old culture were harvested (200 g, 7 min, RT) and resuspended in the same volume of infection medium (Soerensen buffer/HL5 1∶1). Cells were seeded into 25 cm^2^ culture flask and the volume was adjusted to 5 ml with freshly mixed infection medium. The final cell density was 5×10^5^ cells/ml. Before infection the cells were allowed to adhere for 30 min. The medium was removed from the cells and replaced by 5 ml of infection medium with bacteria, multiplicity of infection (MOI) of 10. Following an invasion period of 3 hours (infection time), the remaining extracellular bacteria were killed by a gentamicin treatment (100 µg/ml). After 50 min incubation at 25.5°C, the *Dictyostelium* cells were washed with 5 ml of Soerensen buffer. Then 5 ml of infection medium was added to each flask. For each time point cells were resuspended and 300 µl of the suspension was lysed by centrifugation (7 min, 20,000× g) and vigorous shaking. Serial dilutions of these lysates were plated on BCYE agar.

For the *L. hackeliae* infection serotype 1 was used (ATCC 35250) [70]. The infection assays were done four times in triplicates.

### GST-fusion protein expression and purification

For protein expression *E. coli* BL21 (DE) and XL1 blue were used. Induction of protein expression was induced with 0.25 mM isopropyl β-D-thio-galactoside (IPTG) when an OD_600_ of 0.8 was reached. Cells were further cultured at 30°C for 3 hours. They were harvested, lysed in 50 mM Tris-HCl, pH 7.4 to 8.0, 100 mM NaCl, supplemented with Protease inhibitors (0.5 mM PMSF, 1 mM Benzamidine and Complete (Roche) and 1 mM DTT with an EmulsiFlex cell homogenizer. Lysates were separated into soluble and insoluble fractions by centrifugation at 18,000 g. The fusion proteins from the soluble fraction were purified using GST-Sepharose beads (GE Healthcare).

### Immunofluorescence analysis

Antibodies have been listed in Bakthavatsalam et al. (2007) except for HA-tag antibody 3F10 (Roche Diagnostics, Mannheim, Germany) and mAb 173-185-1 [28]. Fixation of cells was done with methanol (–20°C for 25 min). For labeling acidic compartments LysoTracker Red was used (Invitrogen-Molecular Probes). For live cell imaging cells were monitored using a confocal microscope Leica TCS SP5 (Leica, Wetzlar, Germany).

### Phosphoinositide-binding assay

Phosphoinositide-binding assay using lipid strips supplied by Echelon Biosciences, Inc. (Salt Lake City, Utah, USA) was performed following the protocol of Echelon. Briefly, GST and GST-fusion proteins were eluted from the beads using 20 mM glutathione in TBS-T (50 mM Tris/HCl pH7.2, 100 mM NaCl with 0.2% Tween-20).

The membranes were blocked with 0.1% ovalbumin (Sigma # A-5253) in TBS for one hour at room temperature. After discarding the blocking solution membranes were incubated with 1 µg/ml protein (GST- PIPK_370 – 828_ or GST) in TBS-T at 4°C over night. Then the protein solution was discarded and membranes were washed with TBS-T three times 10 minutes each. Protein binding was detected by western blot analysis with polyclonal GST antibodies as primary and anti-rabbit IgG (Sigma # A-6154) as secondary antibody.

### Nitrogen starvation assay

Nitrogen starvation assay was done as described [71]. Briefly, strains were incubated submerged for one day in FM Medium (ForMedium Ltd, UK). After washing away dead cells the cells were transferred to shaking culture flasks and incubated for two days in FM Medium. Then cells were harvested and washed two times with amino acid free FM Medium. Cells were adjusted to ∼2×10^6^ cells/ml in 20 ml amino acid free FM Medium. Samples were taken at the indicated time points, diluted in Soerensen with 20 mM EDTA and incubated on ice until they were present as single cells. Then serial dilutions were plated on SM plates with *Klebsiella*. After 5 days the *D. discoideum* colonies were counted.

### Phospholipid labeling

A saponin-based cell permeabilization protocol for *Dictyostelium* was adapted to measure phospholipid labeling in wild type (AX2) and *rpkA^−^* cells [37], [72]. Briefly, AX2 cells were developed for 5 h as previously described [73], transferred to still dishes (2.5 cm), allowed to settle to give a confluent monolayer in KK2 (20 mM potassium phosphate buffer, pH 6.1). At regular time intervals, buffer was replaced with labeling solution (139 mM sodium glutamate, 5 mM glucose, 5 mM EDTA, 20 mM PIPES pH 6.6, 1 mM MgSO_4_·2H_2_O, 0,25% (w/v) saponin, 1× phosphatase inhibitor cocktails 1 and 2 (Roche Ltd.), and 1 µCi/ml [γ-^32^P]ATP (Perkin Elmer Ltd.). Following a 6 min incubation, labeling solution was removed, cells were lysed in acidified methanol and phospholipids were separated as previously detailed [74]. Phospholipid labeling was quantified using a Typhoon PhosphorImager. Even loading was determined using total lipid stain with copper sulphate.

### Determination of the endosomal pH:

Endosomal pH was determined according to [34]. Briefly, cells were grown to 2–5×10^5^ cells/ml, harvested and resuspended at a concentration of 3×10^6^ cells/mL in fresh axenic medium and loaded with FITC-dextran (2 mg/ml) (70 000 Mr, Sigma-Aldrich). Basal endosomal pH was measured after loading for 3 h. Cells were collected by centrifugation, washed in 50 mM MES buffer, pH 6.5, then resuspended in 20× MES buffer and the fluorescence intensity was measured using an infinite M 1000 device (Tecan) equipped with Tecan i-control (version 1.6.19.2). The fluorescence excitation ratio (I495/I450) was calculated after subtraction of the background fluorescence. The endosomal pH was then determined from a standard curve.

### Pull down and immunoprecipitation assays

For each pull down and immunoprecipitation experiments 5×10^7^ cells were lysed in 50 mM Tris-HCl, pH 7.4, 100 mM NaCl, 0.5% NP40, supplemented with Protease inhibitors (0.5 mM PMSF, 1 mM Benzamidine and Complete (Roche) by passing them 10 times through a 27 G syringe and 2×20 sec incubation in a sonication bath. Then cells were incubated in agitation (1000 rpm/min) for 15 min at 4°C followed by a centrifugation step at 8,000× g for 5 min. The supernatant was pre-cleared by incubation with protein A beads for 45 min. Pre-cleared lysates were incubated with the indicated antibodies coupled to protein A beads or with GST and GST fusion proteins. After incubation for 3 h or overnight the beads were washed 3× with lysis buffer and the supernatant was completely removed with a Hamilton syringe. The beads were resuspended in 50 µl of SDS-buffer and after incubation for 5 min at 95°C the proteins were separated via SDS-PAGE. Mass spectrometry analysis of co-immunoprecipitated proteins by LC-MS/MS was performed by the CMMC service facilities.

## Supporting Information

Figure S1 **Characteristics of RpkA-HA. (A) RpkA-HA localizes to yeast phagosomes.** 1E7 cells were incubated with TRITC labeled yeast for 15 min and fixed with methanol (−20°C) for 25 min. The cells were incubated with anti-HA-tag antibody 3F10. As secondary antibody goat-anti-rat-IgG conjugated to Alexa 488 was used. Scale bar, 5 µm. **(B) RpkA-HA rescues the developmental phenotype of** ***rpkA^−^*** **cells.** 5×10^7^ cells of Ax2, *rpkA^−^* and of the two rescue strains 1D9 and 1E7 (*rpkA^−^* expressing RpkA-HA) were plated on plates with *Klebsiella* lawn and photographed after 5 days. Scale bar, 1 mm.
(TIF)
