# Supplementary figures and images for: RpkA, a Highly Conserved GPCR with a Lipid Kinase Domain, Has a Role in Phagocytosis and Anti-Bacterial Defense
Source: PLoS One. 2011 Nov 2;6(11):e27311. doi: 10.1371/journal.pone.0027311 (PMC3206951; doi:10.1371/journal.pone.0027311)

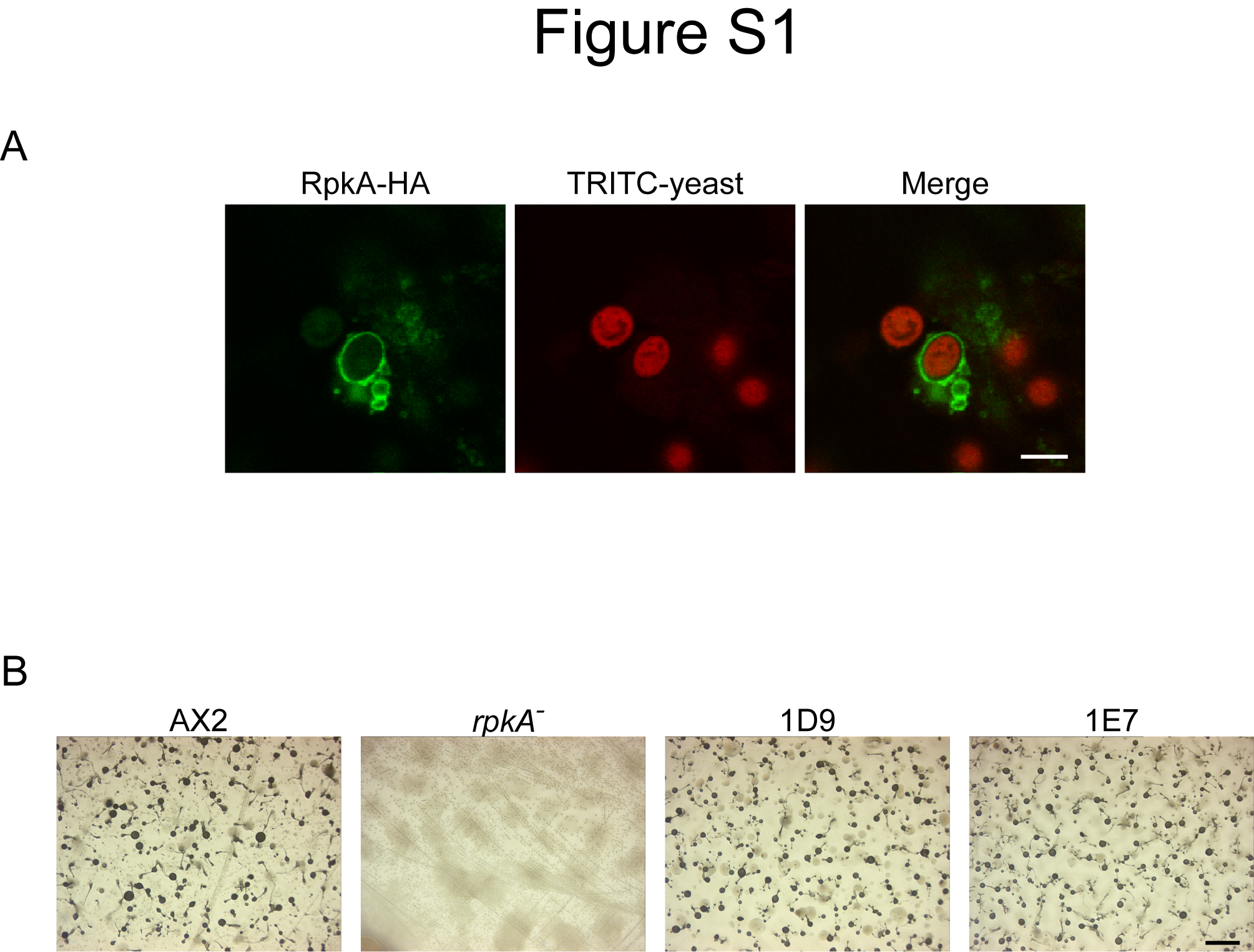

Supplement: Figure S1 — Characteristics of RpkA-HA. (A) RpkA-HA localizes to yeast phagosomes. 1E7 cells were incubated with TRITC labeled yeast for 15 min and fixed with methanol (−20°C) for 25 min. The cells were incubated with anti-HA-tag antibody 3F10. As secondary antibody goat-anti-rat-IgG conjugated to Alexa 488 was used. Scale bar, 5 µm. (B) RpkA-HA rescues the developmental phenotype of rpkA− cells. 5×10^7^ cells of Ax2, rpkA− and of the two rescue strains 1D9 and 1E7 (rpkA− expressing RpkA-HA) were plated on plates with Klebsiella lawn and photographed after 5 days. Scale bar, 1 mm. (TIF) [file pone.0027311.s001.tif]
